# Role of Fimbriae, Flagella and Cellulose on the Attachment of *Salmonella* Typhimurium ATCC 14028 to Plant Cell Wall Models

**DOI:** 10.1371/journal.pone.0158311

**Published:** 2016-06-29

**Authors:** Michelle S. F. Tan, Aaron P. White, Sadequr Rahman, Gary A. Dykes

**Affiliations:** 1 School of Science, Monash University, Bandar Sunway, Selangor, Malaysia; 2 Vaccine and Infectious Disease Organization, University of Saskatchewan, Saskatoon, Saskatchewan, Canada; 3 School of Public Health, Curtin University, Perth, Western Australia, Australia; University of Osnabrueck, GERMANY

## Abstract

Cases of foodborne disease caused by *Salmonella* are frequently associated with the consumption of minimally processed produce. Bacterial cell surface components are known to be important for the attachment of bacterial pathogens to fresh produce. The role of these extracellular structures in *Salmonella* attachment to plant cell walls has not been investigated in detail. We investigated the role of flagella, fimbriae and cellulose on the attachment of *Salmonella* Typhimurium ATCC 14028 and a range of isogenic deletion mutants (Δ*fliC fljB*, Δ*bcsA*, Δ*csgA*, Δ*csgA bcsA* and Δ*csgD*) to bacterial cellulose (BC)-based plant cell wall models [BC-Pectin (BCP), BC-Xyloglucan (BCX) and BC-Pectin-Xyloglucan (BCPX)] after growth at different temperatures (28°C and 37°C). We found that all three cell surface components were produced at 28°C but only the flagella was produced at 37°C. Flagella appeared to be most important for attachment (reduction of up to 1.5 log CFU/cm^2^) although both cellulose and fimbriae also aided in attachment. The *csgD* deletion mutant, which lacks both cellulose and fimbriae, showed significantly higher attachment as compared to wild type cells at 37°C. This may be due to the increased expression of flagella-related genes which are also indirectly regulated by the *csgD* gene. Our study suggests that bacterial attachment to plant cell walls is a complex process involving many factors. Although flagella, cellulose and fimbriae all aid in attachment, these structures are not the only mechanism as no strain was completely defective in its attachment.

## Introduction

Over the past few decades there has been a fast growing and world-wide trend of greater consumption of fresh produce, such as fruits and vegetables, mainly due to a heightened consumer awareness of the benefits of a healthy diet [1,2]. Governments around the world have also encouraged the consumption of fresh produce in an attempt to proactively prevent various diseases such as heart disease, strokes, eye diseases and stomach cancers [3]. The prevalence of foodborne illness associated with consumption of minimally processed produce has, however, also been increasing rapidly [4,5]. Between 1996 and 2005, the consumption of leafy greens in the United States increased by 9% but the incidence of foodborne outbreaks associated with it increased by 39% [6]. Fresh produce is now recognized as the main cause of foodborne outbreaks around the world [7].

It was initially thought that enteric pathogens, which are usually found in the intestinal tracts of animals, would survive poorly on plant surfaces where microorganisms encounter harsh environmental conditions such as drastic temperature fluctuations, desiccation, sunlight and nutrient limitation but recent research [8–11] has shown otherwise. *Salmonella* in particular was previously largely only reported to be associated with foods of animal origin but is now the most commonly identified human bacterial pathogen associated with fresh produce [12,13].

Human foodborne pathogens need to establish themselves on surfaces, including plants, as a precursor to causing foodborne disease and therefore bacterial attachment is a crucial step in their transmission [14,15]. Cut surfaces of plant cell walls (PCWs) are especially vulnerable to the attachment of human foodborne bacterial pathogens as these surfaces lack the waxy cuticle which repels water that could carry pathogens [16,17]. These cut surfaces also exude nutrients and water which are favourable for the growth and survival of the pathogens. Some human pathogens are able to penetrate the internal tissues after attaching to cut plant surfaces which could protect them from chemical sanitizers [18]. Saggers et al. [19] suggested that PCW components at the PCW junction, particularly pectin, may provide receptor sites for *Salmonella* attachment. Our previous findings [20] also showed that pectin alone and pectin in combination with xyloglucan both increased the attachment of *Salmonella* strains.

Studies have found that bacterial cell surface components such as cellulose, flagella and fimbriae are important for the attachment of pathogens to fresh produce [17,21–23]. Flagella are long, thin surface appendages that extend up to 20μm and which are important for motility and chemotaxis [24]. Bacteria use flagella to move along the plant surface before finding a favourable attachment site [25]. Fimbriae are fine, hair-like protein appendages which contain adhesins on their tips with affinity to different sugar molecules and can be up to several micrometers long [26]. Cellulose, which consists of β(1–4)-linked glucose units secreted by bacterial cells, can hinder flagellar rotation and limit bacterial motility [27]. Production of both fimbriae and cellulose are regulated by the *csgD* gene in *Salmonella*. Expression of these structures are associated with biofilm formation and are important for the environmental persistence of *Salmonella* including enhancing its ability to avoid desiccation stress [28,29]. Temperature regulation of the expression of cellulose and fimbriae also takes place at the transcription level of *csgD* [30]. The *csgD* gene was the first enteric bacterial regulator identified to play a more influential role on enteric bacterial interaction with plants than in their animal hosts [10].

The specific mechanisms involved in the association between bacterial cell structures and PCW components exposed on cut PCWs have yet to be elucidated and up to now, very few genetic elements have been identified to be important for the attachment of human foodborne pathogens to plants. However, the ability to form biofilms has been correlated to better survival and stronger attachment to fresh produce [26], for example, *Salmonella* isolates sampled during tomato outbreaks produced biofilms and attached better to tomato leaflets compared to non-biofilm producing strains [31].

No studies reporting the interactions between bacterial surface components and specific PCW components have been reported. Most studies have focused only on the interactions between bacterial components and whole plant tissues. In this study we aimed to investigate how *Salmonella* cell surface structures (flagella, fimbriae and cellulose) influence attachment to major structural components of the PCW (cellulose, pectin and xyloglucan) after growth at 28°C (average environmental temperature) and 37°C (animal and human body temperature). Cases of salmonellosis usually peak during summer in most countries [32]. Most fresh produce have optimal growth temperatures within the range of 21°C to 32°C. For this reason, a growth temperature of 28°C was chosen as it coincides with the average summer temperatures in many countries, including China which is the top global producer of fresh vegetables [33].

In order to investigate this, a bacterial cellulose (BC)-based PCW model was used. The PCW model was produced by culturing *Gluconacetobacter xylinus*, a BC-producing bacterium, in Hestrin and Schramm (HS) growth medium with the addition of pectin and/or xyloglucan. Formation of the PCW model mimics the natural process of PCW deposition in native PCWs [34]. The PCW model was also found to possess similar structural and chemical properties to native PCWs [34,35] and has been previously optimized for studying bacterial attachment to PCWs [36]. Another study [37] has shown that the trend and numbers of *Salmonella* cells attaching to the natural PCWs (potato tuber, apple fruit and lettuce leaves) and to BC composites were similar to each other. In comparison to the heterogeneous composition of native PCWs, the PCW model is more versatile as its chemical composition can easily be manipulated. This allows direct investigation on how bacterial cell surface components interact with individual components in the PCW without the interference of other factors which may complicate the study.

## Materials and Methods

### Bacterial strains and culture conditions

*Gluconacetobacter xylinus* ATCC 53524 and *Salmonella enterica* subspecies *enterica* serovar Typhimurium ATCC 14028 were obtained from the American Type Culture Collection (ATCC; Manassas, VA, USA). Gene knockout mutants of *S*. Typhimurium ATCC 14028 used in attachment experiments and their sources are listed in Table 1. Gene expression in these mutants has been previously well confirmed and characterised [38–40].

**Table 1:** Genotype and characteristics of *S*. Typhimurium ATCC 14028 mutant strains used in this study.

Genotype	Characteristics	Source or reference
Δ*fliC fljB*	Lacks phase 1 and 2 flagellin	Miao et al. [38]
Δ*bcsA*	Lacks cellulose	White et al. [29]
Δ*csgA*	Lacks fimbriae	White et al. [29]
Δ*csgA bcsA*	Lacks cellulose and fimbriae	As described in methods
Δ*csgD*	Missing the major biofilm transcriptional regulator coding sequence, lacks cellulose and fimbriae	MacKenzie et al. [39]

The *G*. *xylinus* strain which produces BC was cultured statically at 30°C for 72h in Hestrin and Schramm (HS) broth containing 2% (w/v) glucose, 0.5% (w/v) peptone, 0.5% (w/v) yeast extract, 0.27% (w/v) Na_2_HPO_4_ and 0.115% (w/v) citric acid [41]. *G*. *xylinus* was maintained on HS agar at 4°C which was prepared by adding 1.5% agar to the HS medium.

For the attachment experiments, wild type and mutant strains of *S*. Typhimurium ATCC 14028 were grown at either 28°C or 37°C in tryptic soy broth (TSB; Merck, Darmstadt, Germany) under shaking incubation (150rpm) (Lab Companion SK-600 benchtop shaker; Medline, UK). Production of cellulose and fimbriae by these strains at 28°C or 37°C were confirmed by monitoring their colony morphology on Luria-Bertani (LB; Merck, Darmstadt, Germany) medium without salt supplemented with 40μg/mL of Congo Red (CR; Sigma-Aldrich, Missouri, USA) and 20μg/mL Coomassie brilliant blue (CBB; Sigma-Aldrich, Missouri, USA) when grown at these two temperatures. Cellulose production was further confirmed using LB medium without salt supplemented with 50μg/mL of Calcofluor White (CW; Sigma-Aldrich, Missouri, USA), colonies which produce cellulose will fluoresce under UV light. Flagella production at both temperatures was determined using Ryu’s flagella stain as described by Kodaka *et al*. [42]. *Salmonella* strains were maintained on tryptic soy agar (TSA; Merck, Darmstadt, Germany) at 4°C.

### Generation of *S*. Typhimurium 14028 Δ*csgA bcsA* double mutant strain

The *S*. Typhimurium ATCC 14028 Δ*bcsA* strain was generated as previously described [29]. *S*. Typhimurium Δ*bcsA* cells were transformed with the pHSG415/Δ*csgA* (formerly Δ*agfA*) construct prepared from S. Enteritidis 27655-3b genomic DNA [43] and selected on LB agar supplemented with 100μg/mL ampicillin. The Δ*csgA* mutation was successfully performed through allelic exchange following established procedures [44]. Final ampicillin-sensitive *S*. Typhimurium Δ*csgA bcsA* colonies were differentiated from Δ*bcsA* colonies by growth at 28°C on agar media (1% tryptone, 1.5% agar) supplemented with 100μg/mL Congo red; Δ*csgA bcsA* colonies appeared light pink, whereas Δ*bcsA* colonies appeared orange or red. PCR was used to confirm the Δ*csgA* mutation, using primers TAFPF (TACGCCAGGAAGGATCAAAACTAT) and TAFPR (GCCGTCGCGCACAGAGA); PCR products were purified and confirmed by DNA sequencing (Eurofins MWG Operon, Kentucky, USA).

### Production of bacterial cellulose-based plant cell wall models

BC-based PCW models were produced as described in our previous paper [20]. Briefly, a primary inoculum of *G*. *xylinus* ATCC 53524 was prepared by transferring a colony grown on HS agar into HS broth which was incubated statically at 30°C for 72h. The primary inoculum was used for the scale-up production of all BC composites and was added to fresh HS medium with or without combinations of pectin and/or xyloglucan as shown below:

- BC was produced with only HS medium without additional additives.
- BC-Pectin (BCP) was produced by adding 0.1%, 0.3% and 0.5% w/v apple pectin (kindly donated by Herbstreith & Fox, Neuenbϋrg, Germany) to the HS medium and an optimal concentration of CaCl_2_ was added to form a low degree of esterification (DE) pectin gel, i.e. 3mM CaCl_2_ for 0.1% w/v pectin, 6mM CaCl_2_ for 0.3% w/v pectin and 12.5mM CaCl_2_ for 0.5% w/v pectin (R&M Chemicals, Malaysia).
- BC-Xyloglucan (BCX) was produced by adding 0.1%, 0.3% and 0.5% w/v xyloglucan (Megazyme, County Wicklow, Ireland) to the HS medium.
- BC-Pectin-Xyloglucan (BCPX) was produced by adding different combinations of pectin and xyloglucan (0.1%, 0.3% and 0.5% w/v), varying concentrations of calcium chloride was added according to the amount of pectin present as shown earlier.

Composites were produced in enclosed plastic containers (1.5cm x 1.5cm x 1.5cm) incubated statically for 72h depending on the HS medium composition. During harvest, BC composites occur as a gelatinous layer floating above the growth medium. Harvested composites were rinsed in 6mM CaCl_2_ at 100rpm for 1h to remove media components. The range of pectin and xyloglucan concentrations were selected based on work carried out previously [20,36,37,45,46] which produced composites with characteristics that fall within average native PCW component concentrations. Chemical composition analysis of the BC composites were consistent when compared to each other [20].

### Attachment to BC composites

Attachment experiments were carried out as described previously [20]. Early stationary phase cultures of *S*. Typhimurium ATCC 14028 and its mutants grown for 18h at either 28°C or 37°C were centrifuged at 5500 x *g* (Hettich D-78532, Tuttlingen, Germany) for 10 min at 4°C. The pellet was washed twice with phosphate buffer saline (PBS) (pH 7.4) (1^st^ BASE, Singapore) and resuspended in PBS to an optical density at 600nm (UV/Vis spectrophotometer, Shimadzu UV mini-1240, USA) which corresponds to 10^8^ CFU/mL for each strain.

After rinsing, BC composites (BC, BCP, BCX, BCPX) (1.5cm x 1.5cm, ~ 2mm thickness) were incubated in 10mL of each pathogenic bacteria suspension (10^8^ CFU/mL) for 20 mins with gentle shaking (100rpm) at 25°C. After incubation, gentle rinsing (100rpm) was carried out in 6mM CaCl_2_ solution for a minute to remove loosely attached cells. Each composite was then placed in a stomacher bag filled with 50mL PBS and pummelled for a minute at 8 strokes/sec in BagMixer 400 (Interscience, France). The resulting stomached fluid was then serial diluted and appropriate dilutions were plated on xylose lysine deoxycholate agar (XLDA; Oxoid, UK) to enumerate the number of pathogenic bacteria attached to the BC composite (CFU/cm^2^ composite). There may be some variability in the surface chemistry of the surface exposed to the air compared to the other surfaces exposed to the HS medium. We have reasonably assumed that this will not significantly affect the attachment results as all surfaces of the composites were exposed to bacteria when the composites were fully immersed in the bacterial suspension.

### Data analysis

All experiments were performed in triplicates with three independently grown bacterial cultures. Statistical analysis of results was performed using Statistical Package for the Social Sciences (SPSS) (PASW Statistics 18, SPSS Inc., USA). One-way analysis of variance (ANOVA) was used to compare significant differences between wild type and mutant strains of *S*. Typhimurium ATCC 14028 grown at the same temperature (either 28°C or 37°C) for their overall attachment to the BC composites. Another one-way ANOVA was carried out individually for each strain grown at a specific temperature (either 28°C or 37°C) to compare significant differences in numbers attaching to different BC composites. Independent sample t-tests were also conducted to determine significant differences for the same strain for its attachment to the BC composites when grown at two different temperatures (comparing 28°C and 37°C). Differences among the means were determined using Tukey’s method at 95% confidence level.

## Results and Discussion

### Production of cell surface components by *S*. Typhimurium ATCC 14028 wild type strains

**Fig 1:**
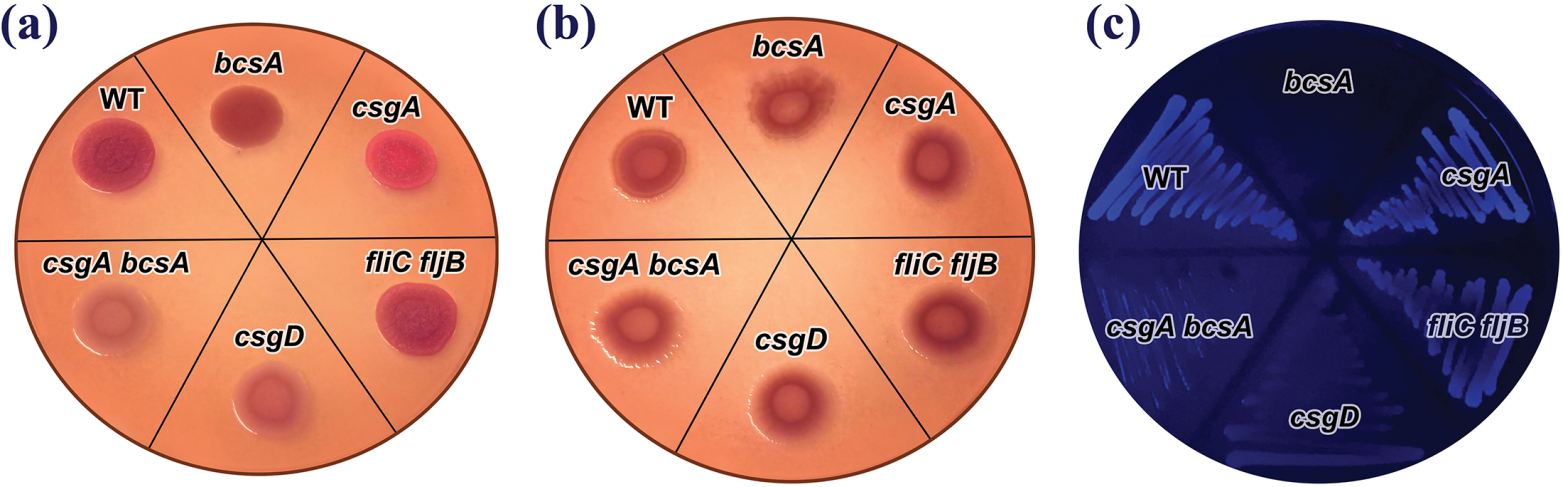
Colony morphology of *Salmonella* Typhimurium ATCC 14028 wild type and mutant strains. Colonies formed on (a) Congo Red (CR) agar plate grown at 28°C, (b) CR agar plate grown at 37°C and (c) Calcofluor White (CW) agar plate grown at 28°C.

*S*. Typhimurium ATCC 14028 produced flagella when grown at 28°C and 37°C (shown in S1 Fig). However, the *S*. Typhimurium strain displayed temperature-dependent expression of cellulose and fimbriae as can be seen on CR and CW plates (Fig 1). As described by Romling et al. [47], the wild type strain of *S*. Typhimurium was able to produce cellulose and fimbriae at 28°C and showed rough, dry and red (rdar) phenotypes on the CR plate. Wild type colonies grown on the CW plate fluoresced under UV light. When grown at 37°C however, the strain lost the ability to produce these structures and appeared as smooth and white colonies (saw) on the CR plate and colonies formed on the CW plate did not fluoresce. White et al. [29] also noted that *Salmonella* only produce these extracellular structures at incubation temperatures of below 30°C and under nutrient-limited conditions at low osmolarity. According to Kader et al. [48], temperature regulation of the rdar morphotype is mediated by the temperature gradient in cyclic-di(3’→5’)-guanylic acid (c-di-GMP) concentrations. The c-di-GMP secondary messenger regulates cellulose and fimbriae production by affecting both CsgA and CsgD expression on the transcriptional and post-transcriptional levels respectively. Therefore, wild type *Salmonella* strains are expected to have lower levels of c-di-GMP at 37°C than at 28°C, this could be caused by either increased phosphodiesterase activity or reduced diguanylate cylase activity [48].

### Effect of PCW components and temperature on *Salmonella* attachment to BC composites

**Fig 2:**
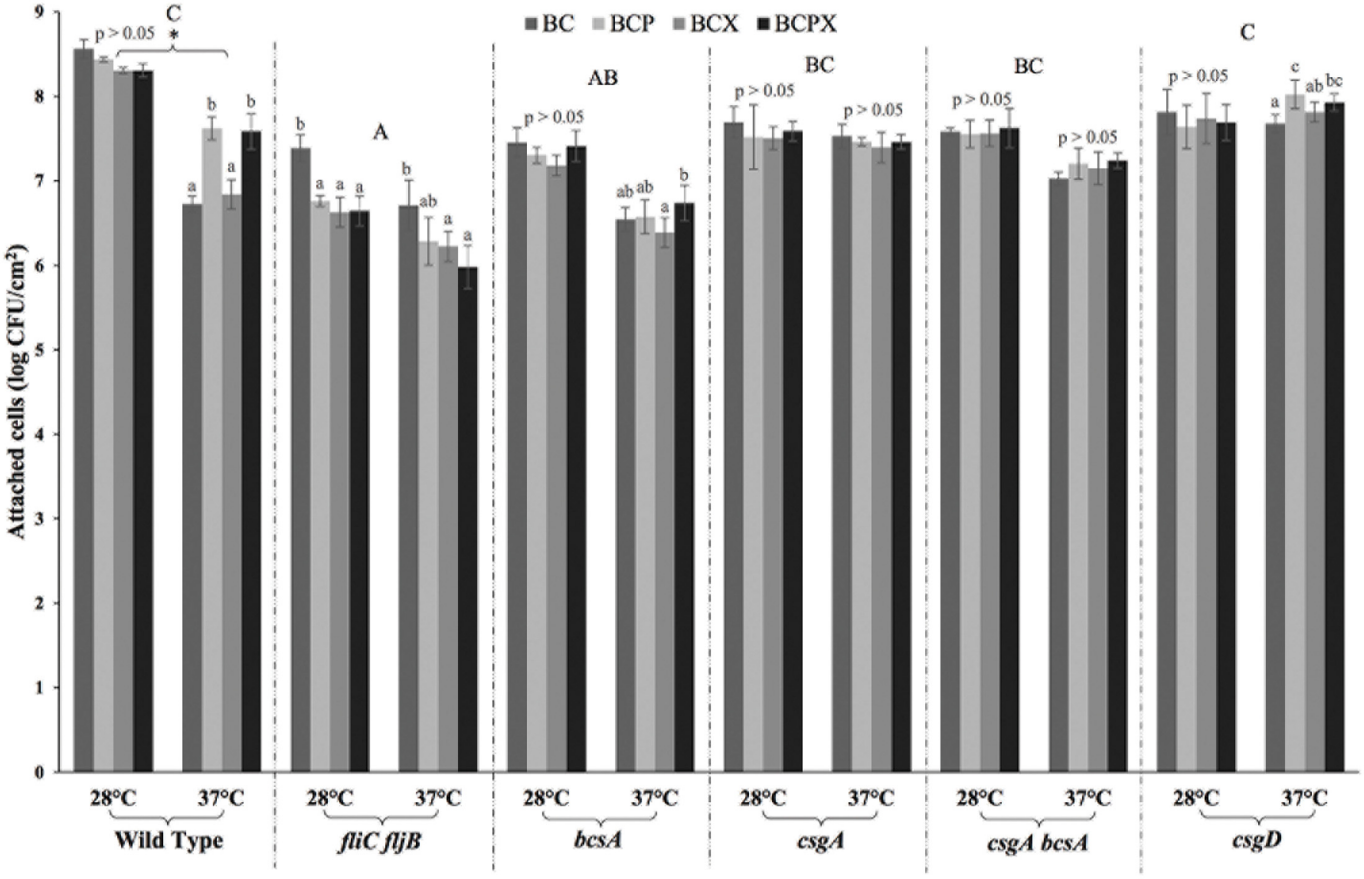
Attachment of *S*. Typhimurium ATCC 14028 (a) wild type, (b) Δ*fliC fljB* mutant, (c) Δ*bcsA* mutant, (d) Δ*csgA* mutant, (e) Δ*csgA bcsA* mutant and (f) Δ*csgD* mutant grown at 28°C and 37°C to various BC composites (BC, BCP, BCX, BCPX). Different lowercase letters indicate significant differences in attachment numbers between different BC composites within each strain grown at a specific temperature (One-way ANOVA & Tukey’s pairwise comparison at p<0.05). Different uppercase letters indicate significant differences in attachment numbers to BC composites between different strains (One-way ANOVA & Tukey’s pairwise comparison at p<0.05). Asterisk sign indicates significant differences in attachment numbers for the same strain grown at two different temperatures (28°C and 37°C) (independent samples t-test at p<0.05).

We found in this study that the varying levels of pectin and xyloglucan (0.1%, 0.3% and 0.5%) did not have significant effect on *S*. Typhimurium attachment to the BC composites (shown in S2 Fig), hence attachment numbers for each type of composite were collated and presented as an average value in Fig 2 for easy comparison. We initially expected the *S*. Typhimurium strains grown at 28°C (especially the wild type strain and the Δ*fliC fljB* mutant which can produce cellulose and fimbriae at 28°C) to have higher attachment levels to the BC composites than the same strains grown at 37°C. However, of the 6 strains only the wild type *S*. Typhimurium strain showed significant difference in the number of cells attached to the BC composites when grown at the two different temperatures (p<0.05) whereas others showed similar attachment for both temperatures (p>0.05). All strains grown at 28°C did not show significant differences in their attachment to the various BC composites (p>0.05) except for the Δ*fliC fljB* mutant which showed the differential attachment to the BC composites. The Δ*fliC fljB* mutant attached at lower levels compared to the other strains and its attachment to the BC composites containing pectin and/or xyloglucan was significantly higher than to the BC-only composite (p<0.05). This suggests that flagella may interact with pectin and xyloglucan, with the loss of flagella decreasing *Salmonella* attachment to the BC composites containing these PCW components. As Warriner and Namvar [49] have pointed out, human pathogen attachment to plants may involve specific recognition interactions between the bacterial cell surface and physical structures on the leaf. Cell surface components (such as cellulose, flagella and fimbriae) may harbour surface epitopes which enable pathogens to preferentially attach to cut surfaces and natural openings, such as stomata, which expose nutrients produced during photosynthesis [12,17]. A study by Saggers et al. [19] also indicated interactions between bacterial cells and PCW components as fewer *S*. Typhimurium cells were attached when less pectin was present in the potato tissue. It was also expected that the attachment for all *S*. Typhimurium strains grown at 37°C (except for the Δ*fliC fljB* mutant) would be similar to each other since cellulose and fimbriae are not produced at this temperature. This was not, however, the case as significant variations in the attachment of different strains were observed (p<0.05). Variations in the attachment of *S*. Typhimurium strains grown at different temperatures were most probably not due to the effect of cellulose and fimbriae structures but could be caused by other factors that have not yet been identified.

### Role of cell surface structures in *Salmonella* attachment to BC composites

An overall comparison on the number of cells of the *S*. Typhimurium ATCC 14028 wild type and mutant strains attached to the BC composites presented in Fig 2 indicated that the different strains attached in significantly different numbers to the composites (p<0.05). It was shown that overall, the Δ*fliC fljB* and Δ*bcsA* mutants displayed significantly lower attachment levels compared to the wild type strain (p<0.05). Another comparison carried out on strains grown at 28°C showed that the wild type strain attached in significantly higher numbers as compared to all mutants (p<0.05). This indicates that cellulose, fimbriae and flagella were all involved in the attachment of *S*. Typhimurium to the PCW models. Lapidot and Yaron [23] suggested that bacterial surface appendages, such as flagella and curli fimbriae, may influence the initial reversible adhesion to plants, which is mediated by van der Waal interactions and hydrogen bonds. This initial adhesion is followed by stronger irreversible attachment which is mediated by electrostatic forces and dependent on extracellular components, such as bacterial cellulose.

#### Role of flagella

Of the bacterial surface structures studied, flagella appeared to have the most important role in attachment as the Δ*fliC fljB* mutant attached in lowest numbers of all mutants to PCW models when grown at both temperatures. As compared to the wild type, attachment of the Δ*fliC fljB* mutant was reduced by ~1.5 and 0.9 log CFU/cm^2^ when grown at 28°C and 37°C, respectively. Flagella are known to be important for the motility of *S*. Typhimurium cells [50] and allow the bacteria to reach the attachment surface faster [51]. Flagella also mediate chemotaxis which can guide planktonic cells to swim towards sites with nutrients or towards cells attached to a surface [52]. Similarly, motility enables pathogens to enter and colonize stomata, wounds and openings in plants [13]. Flagella and motility mutants of *S*. Typhimurium failed to invade lateral root junctions of the *Arabidopsis thaliana* plants, which may be explained by the inability of the mutants to find entry points into the plant [25]. It was also found that mutations which impaired bacterial motility also reduced the ability of *Salmonella* to be internalized by plants [13]. We suggest that flagella enable the cells to move within the matrix of the BC composites where attachment can occur. There is also a possibility that the long flagella filament (which extends up to 20μm) may cause entanglement of *Salmonella* cells (~2μm) within the BC matrix. A study by Berger et al. [22] has shown that *S*. Senftenberg requires flagella to attach to salad leaves. In contrast to the results of our study, these authors found that the deletion of the *fliC* gene did not affect *S*. Typhimurium attachment to basil leaf epidermis. The different outcome of this previous study and ours may be due to the fact that the *fljB* gene was not deleted in the previous study. The normal expression of *fljB* (which encodes the phase-2 flagellin) could have substituted for the loss of phase-1 flagellin (encoded by *fliC*) and thus the ability of the *S*. Typhimurium to attach was not affected in their study. In addition to the use of flagella for motility and chemotaxis, *S*. Typhimurium also uses the flagella to sense external environments in order to regulate its own biogenesis and virulence [53]. It has been demonstrated that the flg22 peptide conserved in the *Salmonella* flagellin activates the plant immune system which then inhibits *Salmonella* colonization. Iniguez et al. [54] showed that the *S*. Typhimurium Δ*fliC fljB* mutant was more successful in colonizing roots in alfalfa, wheat and *Arabidopsis* plants. This could be due to the inability of the plant defence system in detecting colonization by the pathogens. Although the role of flagella in *Salmonella* attachment to PCWs can be studied using the PCW models and cut plant material, these models cannot be used to investigate the plant immune response to human pathogens. Interactions between plants and human pathogens may cause changes in the plant such as the production of free radicals, lignification, pH change and also phenolic and cellulose appositions in the PCW. This represents a limitation of our study and it is necessary to investigate this using native PCWs in future studies to obtain a complete picture of these interactions.

#### Roles of cellulose and fimbriae

In addition to the importance of flagella, our results also showed that cellulose and fimbriae were involved in the attachment of *S*. Typhimurium to PCWs. Single and double mutants of cellulose and fimbriae (Δ*bcsA*, Δ*csgA*, Δ*csgA bcsA*, Δ*csgD*) which were grown at 28°C all displayed significantly lower attachment compared to the wild type strain (p<0.05). When these mutants were grown at 37°C, the Δ*csgA* and Δ*csgA bcsA* attached in similar numbers as compared to the wild type (p>0.05). The Δ*fliC fljB* and *ΔbcsA* mutants attached at significantly lower levels (p<0.05) whereas only the Δ*csgD* mutant showed significantly higher attachment numbers compared to the wild type (p<0.05). The roles of cellulose and fimbriae in human pathogenic attachment to plants are not well understood yet but both attachment structures are known to contribute to aggregative multicellular behaviour, biofilm formation and protection against harsh environmental conditions [55,56].

Fimbriae, which are shorter in length, straighter and more numerous than flagella in numbers do not play a role in *Salmonella* motility. The function of adhesins on the fimbrial tip in determining its specific attachment properties to animal cells has been demonstrated [57]. Lectins, found on fimbriae and flagella structures, are able to recognize oligosaccharide units on the animal cells allowing specific attachment [58]. A similar process could be involved in bacterial attachment to plant surfaces. Cellulose play a role in cell-to-cell interactions and the formation of bacterial aggregates which may in turn favour bacterial attachment to a surface [58,59]. In addition to its role in attachment, cellulose also promotes bacterial persistence in the environment by conferring cells with resistance to adverse conditions, such as exposure to chlorine and bleach [60].

Some studies have shown that cellulose and fimbriae were involved in *Salmonella* attachment to parsley and alfalfa sprout seedlings [23,59]. A number of studies have found that *S*. *enterica* uses cellulose to adhere to plant roots [59,61]. Solomon et al. [62] found that high proportions of *Salmonella* strains isolated from fresh produce possess cellulose and fimbriae, which further supports this finding. Lapidot and Yaron [23] showed that both cellulose and fimbriae affect bacterial colonization although fimbriae played a more important role than cellulose in *S*. Typhimurium transfer from contaminated irrigation water to parsley. Another study by Barak et al. [59] also showed that the roles of cellulose and fimbriae in attachment are additive, with the initial attachment of a Δ*csgB bcsA* double mutant further reduced compared with a Δ*bcsA* single mutant. Barak et al. [21] also observed that mutation in *csgB*, not *csgA*, reduced the ability of *S*. Newport to attach to alfalfa sprouts. They suggested that the curli fimbrial nucleator (encoded by *csgB*) may facilitate the initial attachment of *Salmonella* to plants even without the production of fimbriae.

In our study it appeared that the four cellulose and fimbriae mutants behaved similarly. This feature could be explained by the smaller roles these structures have in bacterial attachment such that the loss of either one or both of these structures did not greatly affect attachment of the strains. To the best of our knowledge, no study has a pleiotropic effect of these gene mutations on the general structure of the bacterial cell wall.

In addition to being involved in the regulation of cellulose and fimbriae expression, the *csgD* gene is also involved in the synthesis of the O antigen capsule and colanic acid which have been shown to modulate bacterial attachment to plants [59]. Colanic acid has been found to be associated with fimbriae in *E*. *coli* and is involved in the formation of *Salmonella* biofilms on animal cells. The O antigen capsule has been shown to protect bacterial cells from desiccation [28]. It was interesting to note that in our study the Δ*csgD* mutant grown at both 28°C and 37°C had significantly higher ability to attach to the composites as compared to the Δ*bcsA* mutant (p<0.05). Studies [63,64] have shown that Δ*csgD* mutants lacking cellulose and fimbriae have increased *fliE* (encoding the flagellum basal body) promoter activity and production of the FliC protein as compared to wild type cells. This can be explained as the synthesis of cellulose and fimbriae or flagella production are mutually exclusive from each other due to opposite regulation by the signalling molecule cyclic di-GMP [65]. Increased production of flagella-related genes could therefore have helped the Δ*csgD* mutant to attach better to the BC composites. Further studies are required to confirm this.

## Conclusions

Taken together the results of our study demonstrate that *S*. Typhimurium cells grown in the animal host (at 37°C) do not produce cellulose and fimbriae and that these biofilm-forming structures will only form if the pathogens are released into the external environment which has a lower temperature (e.g.: 28°C). Flagella, fimbriae and cellulose all contribute to the interaction of *Salmonella* with intact plants in the environment, but we have shown that these structures are not the most important mechanisms for attachment of *Salmonella* to the BC composites which may be influenced by many other factors. Although the results from our study do not fully represent the real life phenomenon occurring on whole plants, a better understanding of the role of bacterial structures on the attachment of *Salmonella* to plants will aid in finding ways to remove pathogens from fresh produce more effectively.

## Supporting Information

S1 Fig Flagella (indicated by arrows) of *S*. Typhimurium ATCC 14028 cells grown at (A) 28°C and (B) 37°C stained with Ryu’s stain. (TIF)

S2 Fig Attachment of wild type and mutant strains of *S*. Typhimurium ATCC 14028 grown at (A) 28°C and (B) 37°C to BC composites with varying levels of pectin and xyloglucan (0.1%, 0.3% and 0.5%). (PDF)
