# Supplementary figures and images for: Role of Fimbriae, Flagella and Cellulose on the Attachment of Salmonella Typhimurium ATCC 14028 to Plant Cell Wall Models
Source: PLoS One. 2016 Jun 29;11(6):e0158311. doi: 10.1371/journal.pone.0158311 (PMC4927157; doi:10.1371/journal.pone.0158311)

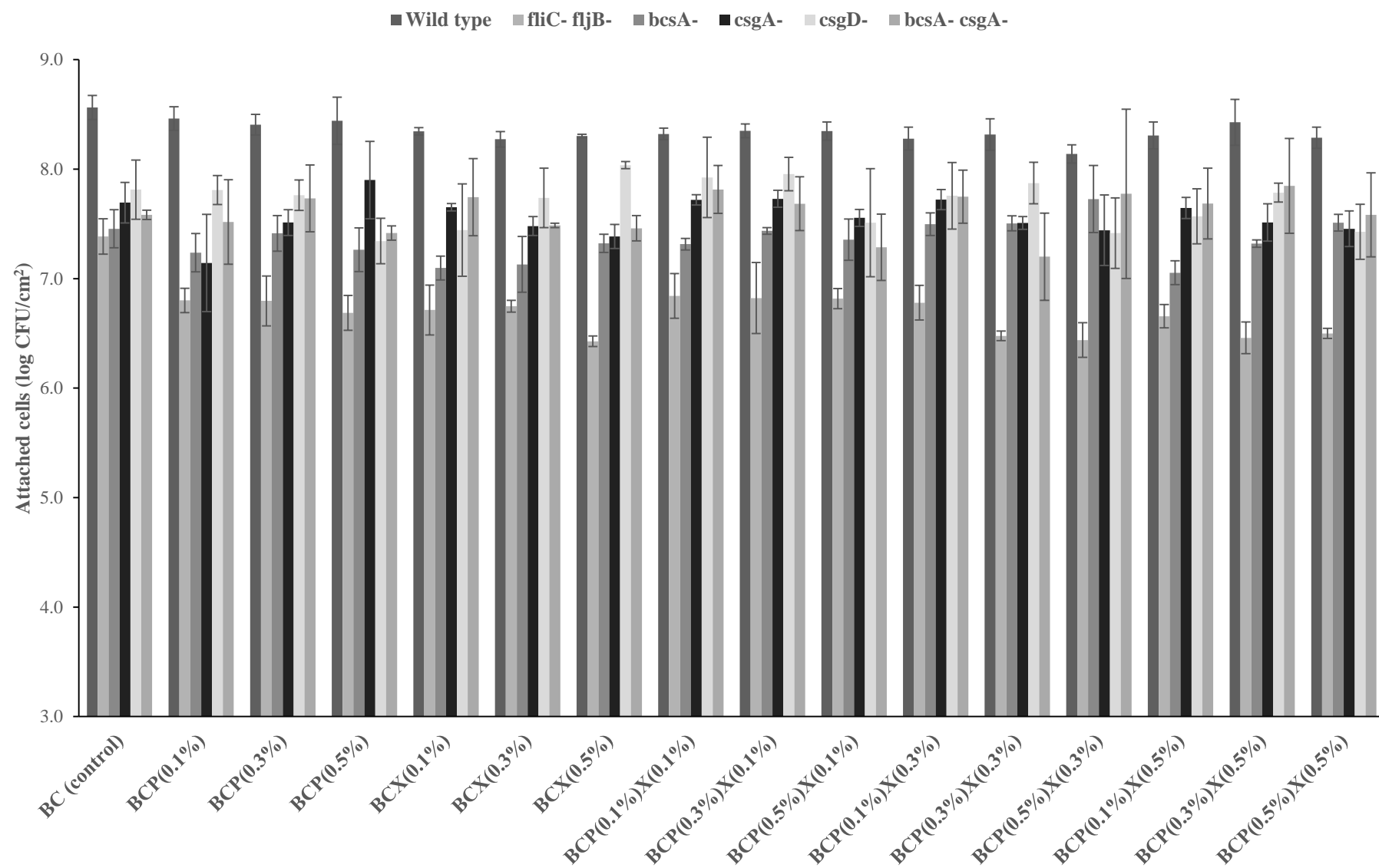

S2 Fig A

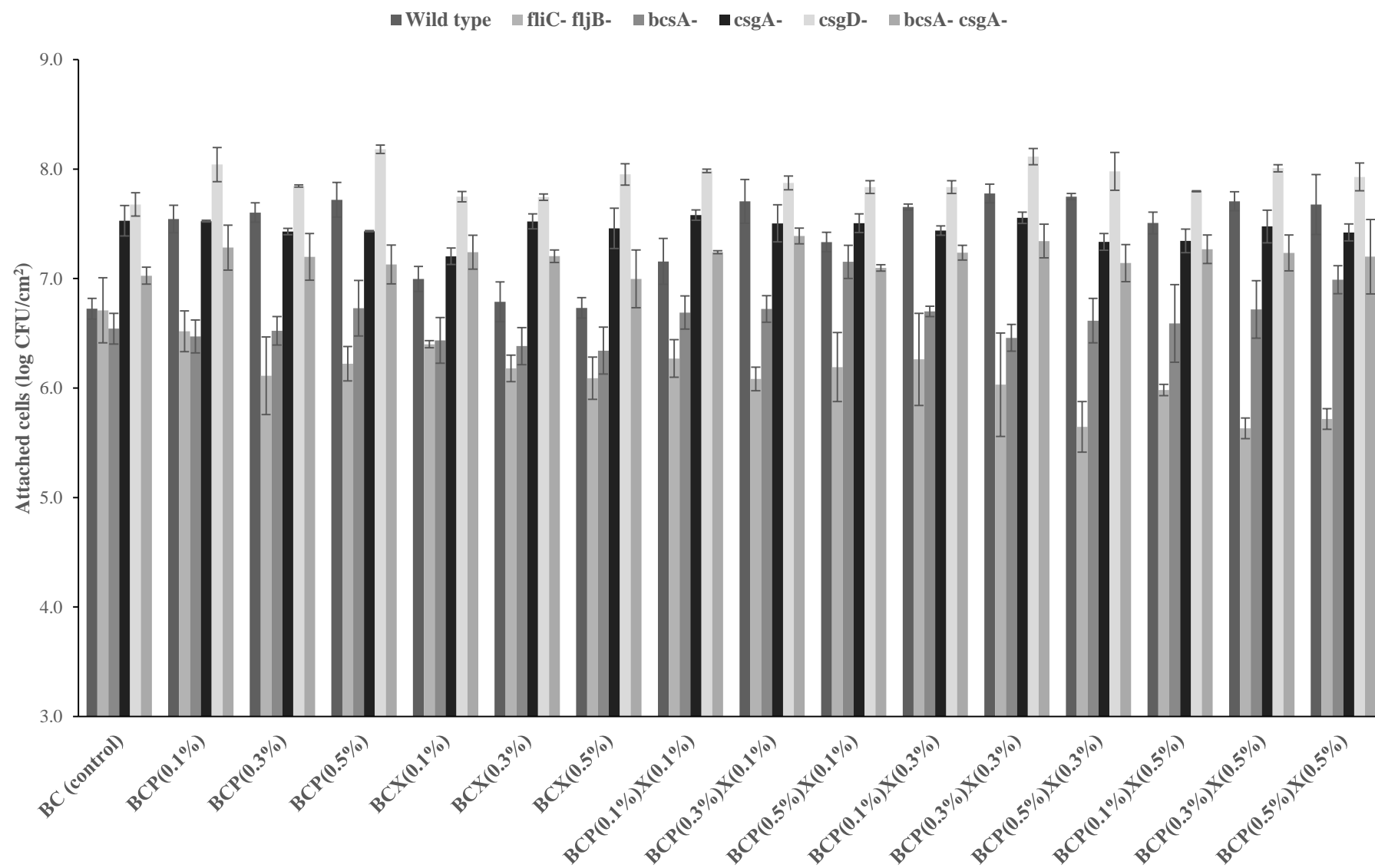

S2 Fig B

Supplement: S2 Fig — (PDF) [file pone.0158311.s002.pdf]
